# Design of a Surface Plasmon Resonance CO Sensor

**DOI:** 10.3390/s22093299

**Published:** 2022-04-26

**Authors:** Francisco Pérez-Ocón, Antonio Manuel Pozo, Jorge Cortina, Ovidio Rabaza

**Affiliations:** 1 Optics Department, University of Granada, 18071 Granada, Spain; ampmolin@ugr.es; 2 Indra Systems S.A., 28108 Madrid, Spain; jcortinad@indra.es; 3 Department of Civil Engineering, University of Granada, 18071 Granada, Spain; ovidio@ugr.es

**Keywords:** CO sensor, surface plasmon resonance sensor, nanosensor

## Abstract

Carbon monoxide (CO) is a highly toxic gas, which can cause death if it is inhaled in small quantities for a long time or in large quantities for a short time. Since this gas can be lethal, it is essential to detect it from minute to large concentrations. Our study consists of the design of a superficial plasmonic resonance (SPR) CO sensor of tiny dimensions which is capable of giving an immediate response at different concentrations. It is designed to work at different heights above sea level since the refractive index of this gas depends on a mixture with air and the air pressure. Due to its low weight and tiny dimensions, it is ideal for space travel or on airplanes. The results show a high resolution and sensitivity (~10^−5^ RIU of resolution and a sensitivity of 13.51–81.26 RIU^−1^).

## 1. Introduction

Automobiles, motorcycles, omnibuses, industry, agriculture, volcanic eruptions, and so on all emit a lot of pollutant gases, amongst them, CO (carbon monoxide). This gas is absorbed by the blood hemoglobin and produces grave respiratory disorders. If it is inhaled in great quantity it can cause death [1,2,3,4,5].

CO is one of the major contaminants of the air emitted directly in many human activities and is responsible for the formation of the tropospheric ozone [6,7].

The presence of CO gas is not perceived by human senses due to its colorless, odorless, and non-irritating nature, therefore accurate and reliable protection devices, such as filters or gas detection sensors, are essential in several fields to prevent the inhalation of CO. This problem is aggravated when there is an escape of this gas in enclosed places (laboratories, homes, means of transports, etc.) and it is also highly flammable.

A micro-cantilever structure made with ZnO was presented to detect CO. A crystal structure of ZnO nanorods was made to grow. The response to gas occurs when the resonance frequency of the micro-cantilever vibration increases. It was capable of detecting picogram levels of CO [8] or detecting concentrations of between 10–50 ppm with a response time of 120 s [9].

In the group of optical sensors, infrared radiation is used and the concentration ranges are very small (100–200 ppm) [10,11,12].

NASA built a sensor for the detection of different gases, specifically, CO (as a product of a fire in its first phase) of low weight for the International Space Station. The system worked with an absorption spectrometer fed with a 4400 nm laser. The average measurement range was 0–500 ppm and the time interval between measurements was 1 s [13].

Other sensors take 10 min to measure a concentration of 100 ppm of CO although the authors affirm that from 20 s, the value of the measurement can already be considered acceptable [14].

With regard to surface plasmon resonance (SPR) sensors for the detection of CO, in 2003, the absorption of plasmons in an Au-CuO layer with wavelengths between 600–800 nm was investigated. It is capable of detecting between 50 and 10,000 ppm of CO in dry air. This fact made it possible to detect CO in the air [15].

Au-YSZ (yttrium stabilized with zirconium) nanocomposite films produce a surface plasmon of around 600 nm in air, and there is a shift towards blue in the presence of CO between 400–500 °C [16].

Lin Kai-Qun et al. [17] showed theoretically that there is a variation of the sensitivity in wavelength interrogation of the surface plasmon of 70 nm (decreasing) with a variation of 70 to 800 K.

An SPR sensor based on nanoparticle sheets of Ag-YSZ, Ag, and Ag-Cu was manufactured. Depending on the incident wavelength, it is possible to detect CO concentrations. With 478 nm, 100 ppm of CO, with 492 nm, 500 ppm of CO, and with 498 nm, 1000 ppm of CO [18].

Ghodselahi et al. [19] designed an SPR sensor with Cu/CuO nanoparticles. They used a wavelength of 600 nm. It was based on the absorption of CO by CuO but the sensor must be cleaned every time the sensor is used.

A system of chemical microsensors was designed together with SPR to detect CO [20]. The method can measure small phase differences for SnO_2_ with different concentrations of CO.

Two sensors were proposed. The first gas sensor was based on monitoring the SPR by introducing NiO and Au nanoparticles into a SiO_2_ matrix. The second is a TiO_2_-Au system within a sol-gel solution [21].

Purkayastha et al. [22], designed an SPR sensor angular interrogation. The sensitivity is 1150 RIU^−1^.

There are some optical sensors that measure CO concentrations in the range of 0.5–100 ppm only at a temperature of 250 °C and it takes from 1 s to measure 0.5 ppm to 1 min to detect a concentration of 100 ppm [23].

All the above SPR sensors have limitations because they are not able to measure all concentrations and at any temperature, pressure, etc.

In the case of SPR sensors, we find concentration problems (all of them measure low concentrations) and there are none that measure by intensity interrogation, therefore, the automation system, if it existed, would be very complex.

In this paper, we propose a plasmonic sensor for measuring CO concentrations by intensity interrogation so that the measurements are continuous and without any moving parts which will be an improvement on the previous ones.

## 2. Design and Simulation of the Plasmonic Sensor

This sensor is based on the principle of surface plasmon resonance. Metals have a charge density due to free electrons. If we apply an external electric field at a place in the metal, the local density of free electrons at that place is changed by the force of the electric field. It is at the metal-dielectric interfaces that plasma oscillations occur. These oscillations are due to the charge density, that is, to the free electrons at the boundary between the metal and the dielectric. The surface plasmon is a quantum of these oscillations, a pseudo particle, and is associated with the transverse magnetic wave (TM) or p-polarized field that has a maximum at the metal-dielectric interface and decays exponentially in both media (in the metal and the dielectric).

To excite a surface plasmon, the wave vector of the excitation radiation at the metal-dielectric interface must be the same as in the surface plasmons (resonance condition).

**Figure 1:**
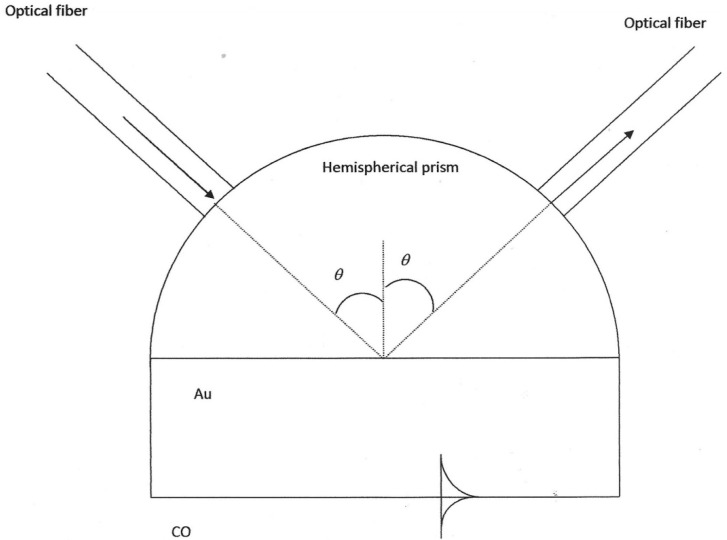
Diagram of the plasmonic sensor. An optical fiber transports the incident radiation on the left part of the hemispherical prism, and another optical fiber collects the reflected radiation on the right. The surface plasmon polaritons are shown propagating along the Au-CO interface.

One way to excite the surface plasmons with an evanescent wave is using the configuration of Figure 1. In this case, the resonance condition is given by:

(1) $$\sin\theta =\sqrt{\frac{\varepsilon_{m}\varepsilon_{s}}{\varepsilon_{p}\left(\varepsilon_{m}+\varepsilon_{s}\right)}}$$

where *ε_m_*, *ε_s_*, *ε_p_* are the dielectric constants of the metal, the medium (CO), and the hemispherical prism, respectively, and *θ* is the angle of incidence (with respect to the normal) at the base of the prism. With these resonance conditions, the energy of the incident radiation is transferred to the surface plasmon and will cause a decrease in the reflected radiation at the interface between the base of the prism and the medium where the CO is. This is valid for any angle of incidence equal to or greater than the critical angle. The operational principle is based on the SPR [24].

Figure 1 shows the scheme of the optical device we plan to design to measure CO concentrations.

The optical fibers do not need focusing elements, they are glued with index equalizing epoxy resin (*n_ep_* = 1.44) to reduce the inevitable Fresnel reflections as much as possible. The optical fibers are glued at an angle with the normal to the base of the hemispherical prism equal to that calculated for each sensor. As it is a hemispherical prism, the light enters perpendicular to its face and we do not have to take into account Snell’s law as is the case of prisms with flat faces. Figure 1 shows the proposed sensor with the Kretschmann configuration. It consists of a hemispherical glass prism from the SUMITA Optical Glass Inc. company (CaFK, *n* = 1.4333) [25], it has a radius of curvature of approximately 0.56 cm with a spherical surface of approximately 4 cm^2^ and a flat surface of 2 cm^2^ (see Figure 1), i.e., a mass of 2.5 g taking into account the density of the CaFK [25].

Taking into account the size of the hemispherical prism, the dimensions of the optical fibers should be: core diameter, cladding, and buffer around 1 mm, 1.05 mm, and 1.25 mm, respectively. The core could be fused silica (glass should be used because plastic optical fibers have a shorter half-life because they degrade earlier) and the cladding could be a polymer. The optical fiber could be a multimode step-index because we do not transport optical encoded signals, and our sensor works with intensity modulation.

The detector must be a high-speed and highly sensitive PIN photodiode chip with, at least, π mm^2^ sensitive circular area to cover the straight section of the output optical fiber. It has to have a peak of sensitivity at 632.8 nm. The minimum accuracy of the detector must be 0.2%, thus the final results do not only depend on the photodetector.

The layer of Au (*n* = 0.12517 + 3.3326i) [26] is 52 nm thick and the optical source is a laser with 632.8 nm of wavelength.

The refractive index of the CO for a wavelength λ = 632.8 nm, from [27] is *n* = 1.00035. If we consider the air-carbon monoxide system as a homogeneous mixture of gases with linear dependence, we can determine the refractive index for each concentration both separately and over the total of the mixture [28]. We can do it in a simple way from the equation below.

(2) $$n=X_{\mathrm{air}}n_{\mathrm{air}}+X_{\mathrm{CO}}n_{\mathrm{CO}}$$

where *X*_air_ and *X*_CO_ indicate the proportions of air and CO over the total mixture and *n*_air_ and *n*_CO_ represent the refractive index of air and CO for the wavelength used.

The method we use to measure CO with this sensor is similar to the one used in [29], which allows us to not confuse the refractive indices of CO with any other gas, although in [29] it is adapted for CO_2_.

We have used the transfer-matrix method to solve the Fresnel equations for the multilayer [30,31] with the WinSpall software package.

The data can be sent in real-time to the base station via wifi, zigbee, 5G, internet, etc., and the results of the CO concentration are displayed. If the base station was not within range, it would be sent to a repeater (radio station) so that finally, linking with different nodes, it would reach the base station. In this way, we can know in real-time the concentration of CO at a remote point.

## 3. Results and Discussion

**Figure 2:**
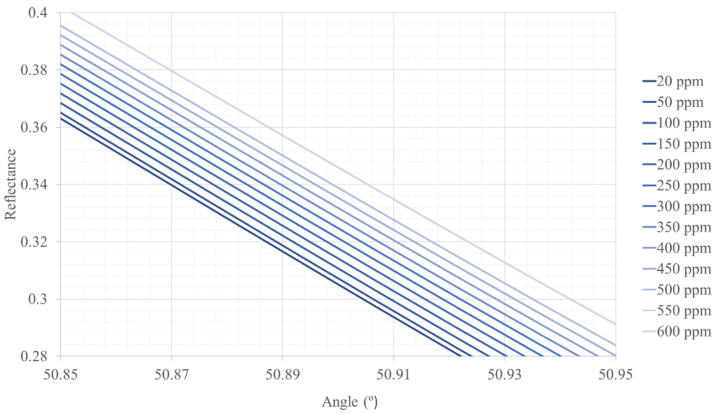
Reflectance curves as a function of the angle of incidence of the light in the hemispherical prism and different concentrations of CO. The height above sea level is 0 m, 101,325 Pa, 25 °C. The sensor works with an incidence angle of 50.91°.

**Figure 3:**
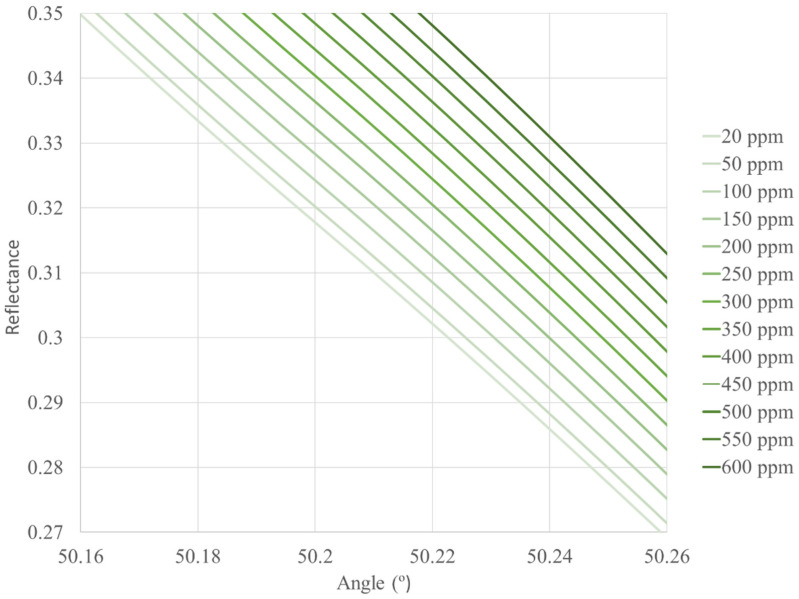
Reflectance curves as a function of the angle of incidence of the light in the hemispherical prism and different concentrations of CO. The height above sea level is 1500 m, 8.45 × 10^7^ Pa, 16 °C. The sensor works with an incidence angle of 50.20°.

**Figure 4:**
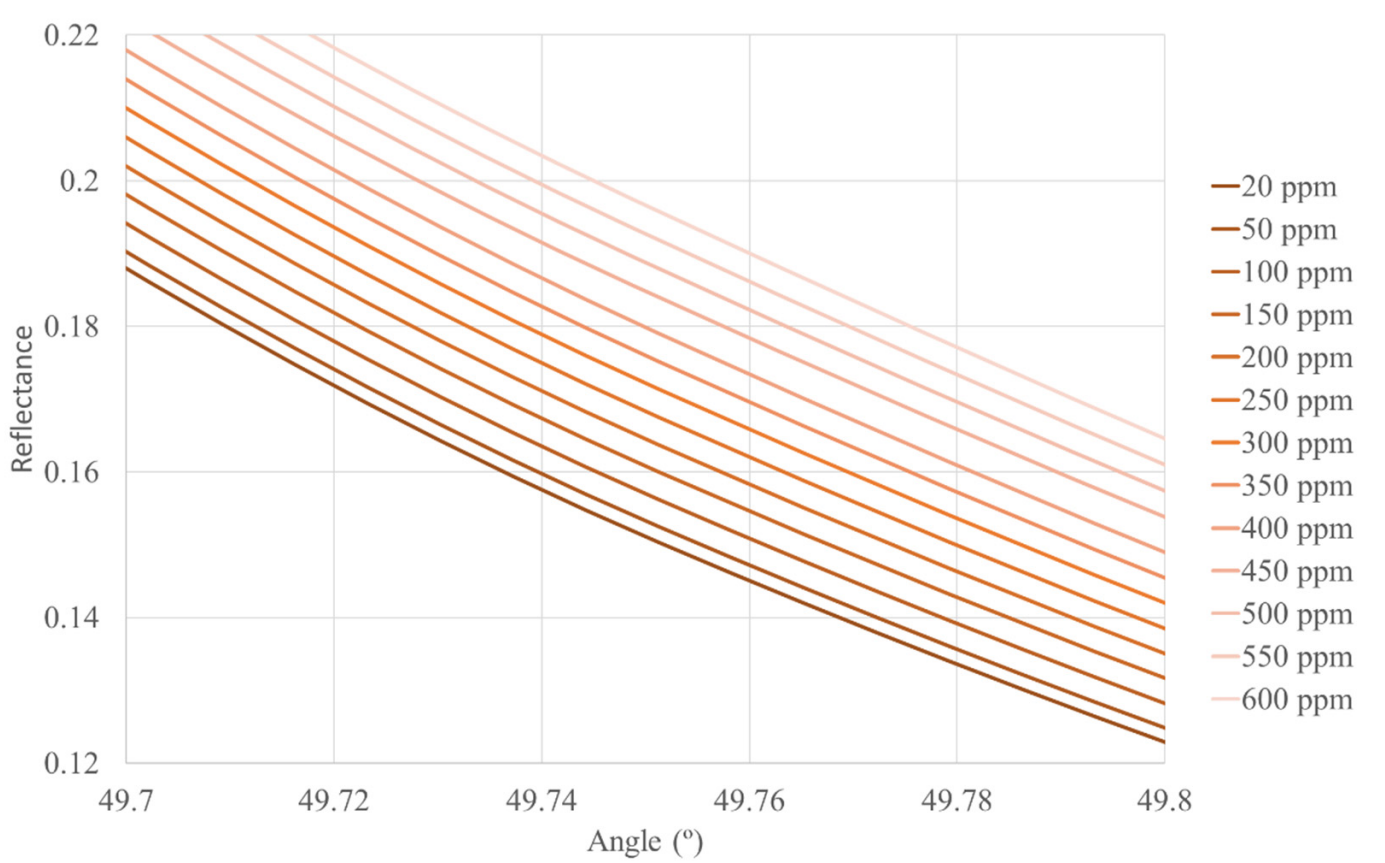
Reflectance curves as a function of the angle of incidence of the light in the hemispherical prism and different concentrations of CO. The height above sea level is 3000 m, 7.01 × 10^7^ Pa, 7 °C. The sensor works with an incidence angle of 49.75°.

Figure 2, Figure 3 and Figure 4 show the reflectance graphics for the different concentrations of CO and for different heights above sea level. By fixing an angle of incidence of the light in the prism for each height, we can calculate the CO concentration (in ppm, for instance) for a given height above sea level from the reflectance recorded by the photodetector.

Table 1 shows the sensitivity and resolution of our sensor for different heights above sea level and different CO concentrations.

We have calculated the reflectance curves from 0–5500 m (5500 m above sea level is the maximum height where trees can grow) intervals of 500 m above sea level, but we only show three figures as an example so as not to increase the length of the paper.

The sensitivity of our sensor is 13.51–81.16 RIU^−1^ and the resolution is 2.46 × 10^−5^–14.80 × 10^−5^ RIU. Depending on the height above sea level (0–5500 m) and the different CO concentrations (0–600 ppm), the refractive index can vary from 1.0002923690 (for 5500 m above sea level and 0 ppm of CO) to 1.000594309 (for 0 m above sea level and 600 ppm of CO). With a variation of the fifth decimal place, we can assure that the measurements of the refractive indexes are reliable enough to measure accurately all the concentrations.

Due to its small volume (2 cm^3^) and mass (5 g), it can be integrated with other devices or be part of the material taken into space, for commercial flights, or indoors or outdoors on the earth.

Table 2 shows the comparison of the different characteristics of CO sensors with ours.

All surface plasmon-based sensors measure in real-time. There are many studies that have proved this, for instance, the research of Homola [36], so our sensor measures in real-time and almost without recovery time. Of the passive materials (hemiprism glass, optical fibers, and Au), the one with the shortest useful life is the optical fiber with about 30 years. The active components (laser and photodetector) are the ones that will decide the useful life of the sensor.

The sensitivity will be the same throughout the life of the sensor because the reflectance measurement is a quotient, that is, a relative measurement, so if the laser emits variable intensities when calculating the quotient, the sensitivity will remain constant.

## 4. Conclusions

We have designed an SPR sensor to determine the amount of CO in ppm capable of measuring in very different environmental conditions. We have obtained different values of reflectance depending on gas concentrations with a resolution of ~10^−5^ and a sensitivity of 13.51–81.16 RIU^−1^. It can measure at any temperature, pressure, and height above sea level as long as the components are not affected. It is the only intensity interrogation sensor published so far. As it has no moving parts, it has no wear or hysteresis whatsoever and is capable of continuously measuring the CO level in real-time and the data can be sent to remote points, also in real-time. Moreover, due to its structure and foundation, it does not need periodic calibrations. It can be part of the material taken to space due to the very small size and weight and could be part of an electronic nose that allows the detection of the presence and quantity of CO since it is an odorless gas. The sensor connected to an antenna, Wi-Fi, ZigBee, etc., is able to send to remote points in real-time.

## Figures and Tables

**Table 1:** Sensitivity and resolution for each height above sea level and each CO concentration.

ppm	0 m above Sea Level		1500 m above Sea Level		3000 m above Sea Level	
	Sensitivity (RIU^−1^)	Resolution (RIU)	Sensitivity (RIU^−1^)	Resolution (RIU)	Sensitivity (RIU^−1^)	Resolution (RIU)
0	57.16	3.47 × 10^−5^	81.35	2.44 × 10^−5^	72.86	2.76 × 10^−5^
20	56.97	3.52 × 10^−5^	81.26	2.45 × 10^−5^	73.15	2.74 × 10^−5^
50	56.31	3.55 × 10^−5^	81.16	2.46 × 10^−5^	73.64	2.72 × 10^−5^
100	55.98	3.57 × 10^−5^	81.07	2.47 × 10^−5^	74.× 10	2.70 × 10^−5^
150	55.57	3.60 × 10^−5^	80.95	2.47 × 10^−5^	74.67	2.68 × 10^−5^
200	55.15	3.63 × 10^−5^	80.81	2.48 × 10^−5^	75.21	2.66 × 10^−5^
250	54.74	3.65 × 10^−5^	80.66	2.48 × 10^−5^	75.73	2.64 × 10^−5^
300	54.32	3.68 × 10^−5^	80.51	2.48 × 10^−5^	76.24	2.62 × 10^−5^
350	53.91	3.71 × 10^−5^	80.34	2.49 × 10^−5^	76.73	2.61 × 10^−5^
400	53.50	3.74 × 10^−5^	80.16	2.50 × 10^−5^	77.19	2.59 × 10^−5^
450	54.74	3.65 × 10^−5^	79.97	2.50 × 10^−5^	77.79	2.57 × 10^−5^
500	54.32	3.68 × 10^−5^	79.76	2.51 × 10^−5^	78.38	2.55 × 10^−5^
550	53.91	3.71 × 10^−5^	79.57	2.51 × 10^−5^	78.76	2.54 × 10^−5^
600	53.50	3.74 × 10^−5^	79.35	2.52 × 10^−5^	79.16	2.53 × 10^−5^

**Table 2:** Summary of the comparison/discussion of the CO sensors with ours.

Sensitivity	Measure Range (ppm)	Response Time (s)	Resolution	Size	Main Compounds
Low [32]	0–100 [33]	14 s and a recovery time of 50 s [34]	Low [31]	Large [13]	Au-CuO [15,35]
Our sensor higher sensitivity	50 [34]	Our sensor measure in real-time	Our sensor higher resolution		Au-YSZ [16]
	0.1–500 [13]				Ag, Au, and Ag-Cu [18]
					Hexagonal array of Cu@CuO core-shell nanoparticles on the a-C:H thin film [19]
					Graphene [22]
					ZnO [23]
					Our sensor only Au, simplest

## Data Availability

Not applicable.
